# Complement in the Pathophysiology of the Antiphospholipid Syndrome

**DOI:** 10.3389/fimmu.2019.00449

**Published:** 2019-03-14

**Authors:** Shruti Chaturvedi, Robert A. Brodsky, Keith R. McCrae

**Affiliations:** ^1^Division of Hematology, Department of Medicine, Johns Hopkins University School of Medicine, Baltimore, MD, United States; ^2^Hematologic Oncology and Blood Disorders, Taussig Cancer Institute, Cleveland, OH, United States; ^3^Department of Cellular and Molecular Medicine, Cleveland Clinic, Cleveland, OH, United States

**Keywords:** antiphosholipid antibodies, complement, thrombosis, endothelial, beta2 - glycoprotein I

## Abstract

The antiphospholipid syndrome (APS) is characterized by thrombosis and pregnancy morbidity in the presence of antiphospholipid antibodies (aPL). Complement is a system of enzymes and regulatory proteins of the innate immune system that plays a key role in the inflammatory response to pathogenic stimuli. The complement and coagulation pathways are closely linked, and expanding data indicate that complement may be activated in patients with aPL and function as a cofactor in the pathogenesis of aPL-associated clinical events. Complement activation by aPL generates C5a, which induces neutrophil tissue factor-dependent procoagulant activity. Beta-2-glycoprotein I, the primary antigen for pathogenic aPL, has complement regulatory effects *in vitro*. Moreover, aPL induce fetal loss in wild-type mice but not in mice deficient in specific complement components (C3, C5). Antiphospholipid antibodies also induce thrombosis in wild type mice and this effect is attenuated in C3 or C6 deficient mice, or in the presence of a C5 inhibitor. Increased levels of complement activation products have been demonstrated in sera of patients with aPL, though the association with clinical events remains unclear. Eculizumab, a terminal complement inhibitor, has successfully been used to treat catastrophic APS and prevent APS-related thrombotic microangiopathy in the setting of renal transplant. However, the mechanisms of complement activation in APS, its role in the pathogenesis of aPL related complications in humans, and the potential of complement inhibition as a therapeutic target in APS require further study.

## Introduction

Antiphospholipid syndrome (APS) is a systemic autoimmune disorder characterized by thrombosis affecting the venous or arterial vascular systems, and/or obstetrical morbidity along with the persistent presence of antiphospholipid antibodies (aPL), including lupus anticoagulant, anticardiolipin antibody and anti-beta-2-glycoprotein-I (β2GPI) (1). Rather than binding to anionic phospholipids such as cardiolipin as was previously believed, aPL are directed against phospholipid binding proteins bound to an appropriate biological surface such as a cellular membrane (2). Anti-β2GPI antibodies are the primary pathogenic antibody in APS (3–5), although aPL directed against other antigens such as prothrombin and phosphatidylserine have also been described (3, 6). The mechanisms by which aPL induce thrombosis and pregnancy loss are not fully understood. Though β2GPI is the primary antigen in APS, its inhibition does not directly have thrombotic effects as evidenced by the lack of a thrombotic phenotype in β2GPI deficient individuals (7, 8). Multiple pathogenic mechanisms have been proposed including inhibition of the natural anticoagulant and fibrinolytic systems (9–12), activation of vascular cells including endothelial cells (13), platelets (14), and monocytes (15), procoagulant effects of extracellular vesicles (16), and disruption of the anticoagulant annexin A5 shield on cellular surfaces (17). Over the past two decades, complement activation has emerged as an attractive target for mechanistic and therapeutic investigations based on studies demonstrating evidence of complement activation in patients with APS, and murine models that indicate a critical role of complement in aPL-mediated thrombosis (18–21) and obstetric (22–24) complications (25). It remains unclear whether these are distinct mechanisms that reflect antibody heterogeneity or are linked to an as yet undefined central mechanism. Recurrent thrombosis and fetal loss are common despite standard treatment. The variability of treatment effectiveness in APS suggests that subsets of patients might benefit from treatments beyond anticoagulation.

**Figure 1:**
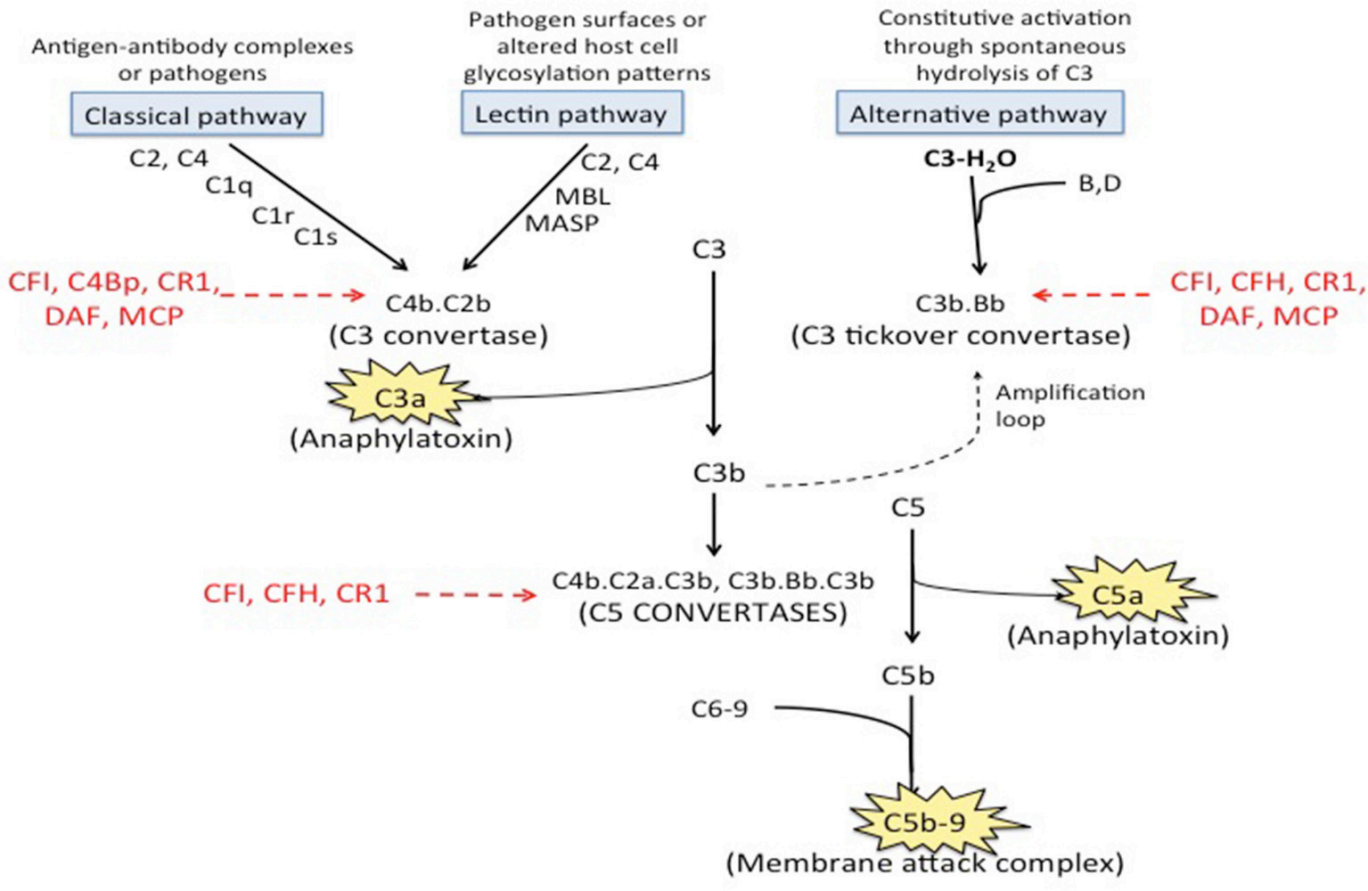
Complement pathways. There are three well-recognized pathways of complement activation; (1) the classical pathway, (2) the lectin pathway, and (3) the alternative pathway. The classical and lectin pathways are activated when specific triggers are recognized by host pattern-recognition receptors while the alternative pathway is constitutively active. Activation of all there pathways ultimately leads to generation of a C3 convertase (C4b.C2a for the classical and lectin pathways and C3b·B for the alternative pathway), which cleave C3 to generate C3a and C3b. C3a is an anaphylatoxin. C3b is quickly inactivated when it lands on a healthy host cell but triggers a rapid amplification loop when it binds to a pathogen or altered host cell. C3b also complexes with the C3 convertases to form the C5 convertases (C4b·C2a·C3b and C3b·Bb·C3b) that cleave C5 into C5a (an anaphylatoxin) and C5b. C5b combines with C6-9 to form C5b-9, also called the membrane attack complex (MAC). Regulatory factors including decay accelerating factor (DAF, CD55), CD59, factor H (CFH), factor I (CFI), membrane cofactor protein (MCP) and C3b/C4b receptor 1 (CR1) act at various stages of the cascade to control complement activation.

The complement system, consisting of over 50 plasma proteins involved in host defense, is organized into three pathways; the immune complex mediated classical pathway, the lectin pathway, and the alternative pathway. These pathways converge at the level of complement component C3 and the terminal complement pathway that leads to generation of C5a, a potent pro-inflammatory molecule, and C5b-9 (the membrane attack complex) (Figure 1). Anti- β2GPI antibodies are associated with complement activation, and the complement and coagulation pathways are closely linked (26, 27); C5a induces neutrophil tissue factor-dependent procoagulant activity (26) and may inhibit fibrinolysis though increased activation of thrombin activated fibrinolysis inhibitor (TAFI). C3a and C5a also activate endothelial cells, inducing the expression of adhesion molecules and procoagulant activity (28–30), as well as platelets (31). Complement activation also causes placental inflammation and injury (32), a hallmark of fetal loss in APS (23). This review summarizes the current evidence supporting the role of complement in aPL associated clinical events, the interplay between complement and thrombosis in APS, therapeutic perspectives on complement targeted agents in APS, and areas of future research.

## Complement in Obstetric APS

### Animal Models of Obstetric APS

The earliest and most compelling evidence for complement involvement in APS comes from murine models of aPL-induced pregnancy loss in which Branch et al. showed that passive transfer of IgG fractions from patients with aPL led to fetal loss (33). On histopathologic examination, IgG localized to the decidua, which showed prominent necrosis. A subsequent series of experiments confirmed this finding and elucidated the role of complement in aPL induced fetal loss. *In vivo* experiments have shown that β2GPI localizes to the decidua (34), and that aPL binding to β2GPI inhibits trophoblastic proliferation, syncytia formation and invasion into maternal decidua, which are required for successful placentation (35).

Holers et al. showed that intraperitoneal injection of IgG from patients with APS into pregnant mice led to fetal resorption in 40% of pregnancies and a 35% fetal weight reduction compared with control mice (36). Inhibition of the complement cascade with the C3 convertase inhibitor complement receptor 1–related gene/protein y (Crry)-Ig prevented aPL mediated fetal resorption. C3 deficient mice (C3^−/−^) were also resistant to aPL mediated fetal loss (36). Girardi et al. later demonstrated that C5 deficiency or treatment of mice with anti-C5a monoclonal antibody protects against aPL induced pregnancy loss and growth retardation (22). Placentae from the aPL IgG treated mice showed human IgG deposition in the decidua, which demonstrated focal necrosis and apoptosis with neutrophil infiltrates (36). Neutrophils recruited by C5a expressed tissue factor that potentiated neutrophil activation and the respiratory burst leading to trophoblastic injury and fetal loss (24, 32). The absence of aPL-induced growth retardation and fetal resorption in mice deficient in C4 or C5 suggests that the classical pathway is involved in initiating these effects. However, factor B is necessary for aPL mediated fetal loss and its inhibition ameliorates these effects supporting a role of the alternative pathway in amplifying complement activation (37). Taken together, these studies suggest that C3 and C5 activation is central to aPL-mediated fetal loss in this model, with neutrophils and tissue factor playing pro-inflammatory roles. Girardi et al. have also suggested that the protective effect of heparin in APS pregnancies may reflect its inhibitory effects on complement (23).

### Complement Activation in Human Studies of Obstetric APS

Studies in humans support the role of complement in aPL mediated pregnancy complications. Hypocomplementemia, suggesting complement activation, has been observed in patients with SLE and APS (38), as well as those with primary APS and obstetric complications (39–41); however others have not found an association with hypocomplementemia and pregnancy complications in APS (42). In the PROMISSE study, which included nearly 500 pregnant women with lupus and/or aPL, adverse pregnancy outcomes were associated with increased serum levels of complement products Bb and C5b-9 early in pregnancy (43). In addition to elevated levels of complement activation products in serum, C4d was deposited at the feto-maternal interface in the placentae of women with SLE or APS, and correlated with fetal loss, decidual vasculopathy, increased syncytial knots and villous infarcts (44, 45). Interestingly, C5b-9 deposition in the trophoblast was not increased compared with control placentae, leading the authors to suggest that C5b-9 may not play a central role in aPL mediated placental injury, which is more likely to be caused by C3a and C5a mediated inflammation (45). Overall, these findings support a role for complement in aPL mediated pregnancy complications; however, the exact mechanisms of complement activation remain to be determined.

## Complement in Vascular APS

### Animal Models of Thrombotic APS

Animal models of thrombotic APS support a role for complement in aPL mediated thrombosis. Most early models of aPL induced thrombosis included passive transfer of aPL along with direct vessel injury by pinching (19, 46) or other means to induce thrombosis, which was reduced in mice with deficiencies of complement proteins C3, C5, or C6 (19), or in the presence of an inhibitory antibody against C5 (18). However, mechanical or chemical endothelial injury to initiate thrombosis that is propagated in the presence of aPL differs from the usual events in APS, in which a localized vascular insult is usually absent. Fischetti et al. used rats primed with lipopolysaccharide, which does not cause thrombosis by itself (20). Administration of aPL IgG to LPS primed mice led to thrombosis while administration of control IgG did not. Intravascular microscopy showed thrombosis in mesenteric vessels, and immunofluorescence staining confirmed co-localization of IgG and C3 in the vessel wall (20). Thrombosis was markedly attenuated in C6 deficient (C6^−/−^) rats or animal treated with a C5 inhibitor (20). In another set of experiments, a recombinant single-chain fragment variable recognizing domain 1 of β2GPI induced thrombosis in wild type male Wistar rats primed with lipopolysaccharide and pregnancy loss in female mice, but these effects were blocked in C6 deficient rats or C5 depleted mice (21). A CH2 deleted version of this antibody still recognized β2GPI but failed to fix complement and did not induce thrombosis or pregnancy loss. In addition to demonstrating the critical role of complement in aPL induced thrombosis, these experiments show that unlike effects of anti-β2GPI on the placenta, the procoagulant effects of aPL require a priming factor or “second hit” provided by an inflammatory stimulus such as lipopolysaccharide (34). In these murine models of thrombotic APS, C9 is deposited on the vascular endothelium indicating the presence of the membrane attack complex (20, 21). The membrane attack complex triggers the extrinsic pathway of coagulation by inducing tissue factor expression on the endothelial surface (47).

### Complement Activation in Patients With APS

A role for complement activation in patients with thrombotic APS was first suggested nearly 25 years ago by the demonstration of higher serum levels of C5b-9 in patients with aPL and stroke compared with non-APS related stroke (48). Others have reported hypocomplementemia (39) and higher levels of complement fragment Bb and C3a (40, 49) in patients with APS; however, the association with APS-related thrombotic events or serologic characteristics is inconsistent (39, 49). More recently, deposits of C1q, C4, C3, and C5b-9 were noted to co-localize with β2GPI and IgG in the affected artery wall of a patient with primary APS and arterial thrombosis who also had increased plasma levels of C5a and C5b-9 (50).

A minority (1%) of patients with aPL develop catastrophic APS (CAPS), manifesting as small vessel thrombosis in three or more organs within the span of a week in the absence of small vessel inflammation on histopathologic examination (51); CAPS is fatal in over 40% of cases (52). Increased serum C5b-9 has been detected in CAPS with clinical improvement after treatment with eculizumab correlating with a reduction in serum C5b-9 and increase in C3 and C4 (53). While animal studies and anecdotal success of complement inhibitory therapy support a role for complement in aPL induced thrombosis (Table 1) (50, 54, 61), there is a paucity of controlled clinical research studies, and the mechanisms of complement activation in APS and its correlation with vascular events remains incompletely understood.

**Table 1:** Reports of Eculizumab therapy for patients with catastrophic APS or severe APS.

	**Patient**	**Prior therapies**	**Eculizumab dose/duration**	**Outcome**
Shapira et al. (54)	28/M with SLE and APS with a pulmonary embolism at age 12, and arterial ischemia leading to leg amputation, mesenteric ischemia and recurrent CAPS	Heparin, argatroban, fondaparinux, cyclophosphamide, steroids, intravenous immunoglobulin, lepirudin, bivalirudin, aspirin, and clopidogrel, plasma exchange	Eculizumab, 900 mg, then 1,200 q 2weeks for 1 year	Resolution of anemia, thrombocytopenia, and thrombotic events
Appenzeller et al. (55)	30/F with ITP and primary APS developed CAPS after pregnancy. Complicated by myocardial infarction and renal failure	Hydroxychloroquine, heparin, steroids, rituximab, plasma exchange, immunoadsorption, hemodialysis	Eculizumab × 3 months, mycophenylate, steroids (homozygous for C3 mutation, c.1677C>T; p.C559C)	Resolution of MAHA and thrombocytopenia. Later had partial relapse, dialysis dependent
Muller-Calleja et al. (56)	3 patients undergoing renal transplant, 2 with prior CAPS	Prednisone, rituximab, anticoagulation	Eculizumab, 900 mg weekly begun the day after transplant, then 1,200 q 2 weeks	Successful engraftment up to 4 years, continued treatment
Strakhan et al. (57)	36/F with hypertension, acute renal failure, strokes, acute coronary syndrome, and MAHA	Plasma exchange, steroids	Eculizumab 900 mg/wk × 4 then 1,200 q 2 weeks	Gradual improvement of MAHA, continued dialysis
Wig et al. (58)	47/M with APS, multifocal thrombi, and thrombocytopenia followed by renal and liver infarcts	Heparin, plasma exchange, intravenous immunoglobulin, steroids, argatroban, heparin	Eculizumab 900 mg × 2 weekly doses, then 1,200 mg every 7–10 days	Gradual improvement in all parameters, but remains dialysis dependent
Gustavsen et al. (59)	22/F with arterial thrombosis and ischemic ulcerations during pregnancy	Warfarin, low molecular weight heparin, aspirin	Eculizumab 600 mg × 2 weekly doses, prior to Cesarean section	Improvement of ischemic pain, no further thrombosis, no adverse fetal effects
Marchetti et al. (60)	33/F with factor V Leiden and triple positive APS developed TMA at 30 weeks of gestation	Rituximab, aspirin, heparin	Eculizumab 600 mg, Cesarean section at 32 weeks, repeat Eculizumab after surgery	Stabilization of thrombocytopenia, renal function and hematocrit

## Mechanisms of Complement Activation in APS

The aPL profile can predict risk of thrombosis and pregnancy morbidity. For example, LA was associated with a higher risk of thrombosis (OR 3.6, 95% CI 1.2–10.9) than anti-β_2_GPI (OR 2.4, 95%CI 1.3–4.2) and anti-prothrombin (anti-PT) antibodies (OR 1.4, 95% CI 1.0–2.1) in the Leiden thrombophilia study (62). Retrospective and prospective studies have not shown a consistent association between thrombosis and aCL (3, 63). However, the differential ability of different aPL to activate complement has not been studied extensively. Since β_2_GPI is the primary antigen in APS, the anti-β_2_GPI antibody has been proposed as the more clinically significant and predictive aPL (62, 64, 65). Available studies highlight the role of β_2_GPI as a complement regulator (66), though others suggest a role of anti-C1q antibodies (67).

The mechanisms of complement activation in APS are not fully understood. Some have suggested that immune complexes in APS bind to C1q and activate the classical complement pathway (68). However, aCL and anti-β2GPI are frequently of the IgG2 subclass (69, 70), which has relatively weak ability to activate complement compared with IgG1. Anti-C1q antibodies have been detected in patients with SLE, in whom they correlate with clinical manifestations particularly lupus nephritis (71, 72). In a mouse model, an anti-C1q monoclonal antibody enhanced complement activation by the classical pathway and caused renal injury (67). Oku et al. (73) have reported that antibodies against C1q were more prevalent in patients with primary APS (36%) than controls with other non-SLE autoimmune disorders, and concluded that these antibodies contribute to complement activation, since titers of anti-C1q correlated with levels of C4a. However, anti-C1q antibodies did not correlate with thrombosis or pregnancy loss although a small subset of patients with recurrent thrombosis had a higher rate of anti-C1q antibodies (73). Finally, there is essentially no data on activation of complement by IgM anti-β2GPI antibodies.

Gropp et al. have reported a complement regulatory role of β2GPI, which inhibits complement activation by enhancing C3/C3b degradation (66). It has been proposed that when bound to a surface, β2GPI undergoes a conformational change from a circular form to an elongated form that can bind C3; in turn, C3 undergoes a conformational change to expose binding sites that make it susceptible to degradation by complement factor H (CFH) and factor I (66). In addition to its inhibitory effects on complement activation, CFH also has structural similarity to β2GPI and appears to share its property of inhibiting of contact pathway activation triggered by anionic phospholipids (74). Some APS patients, particularly those with recurrent thrombotic events, have autoantibodies against CFH suggesting a role of these antibodies in the predisposition to thrombosis (75, 76). A recent study reported low levels of CFH in patients with primary APS who also had low C3 suggesting complement activation (77).

## Complement and Vascular Thrombosis

**Figure 2:**
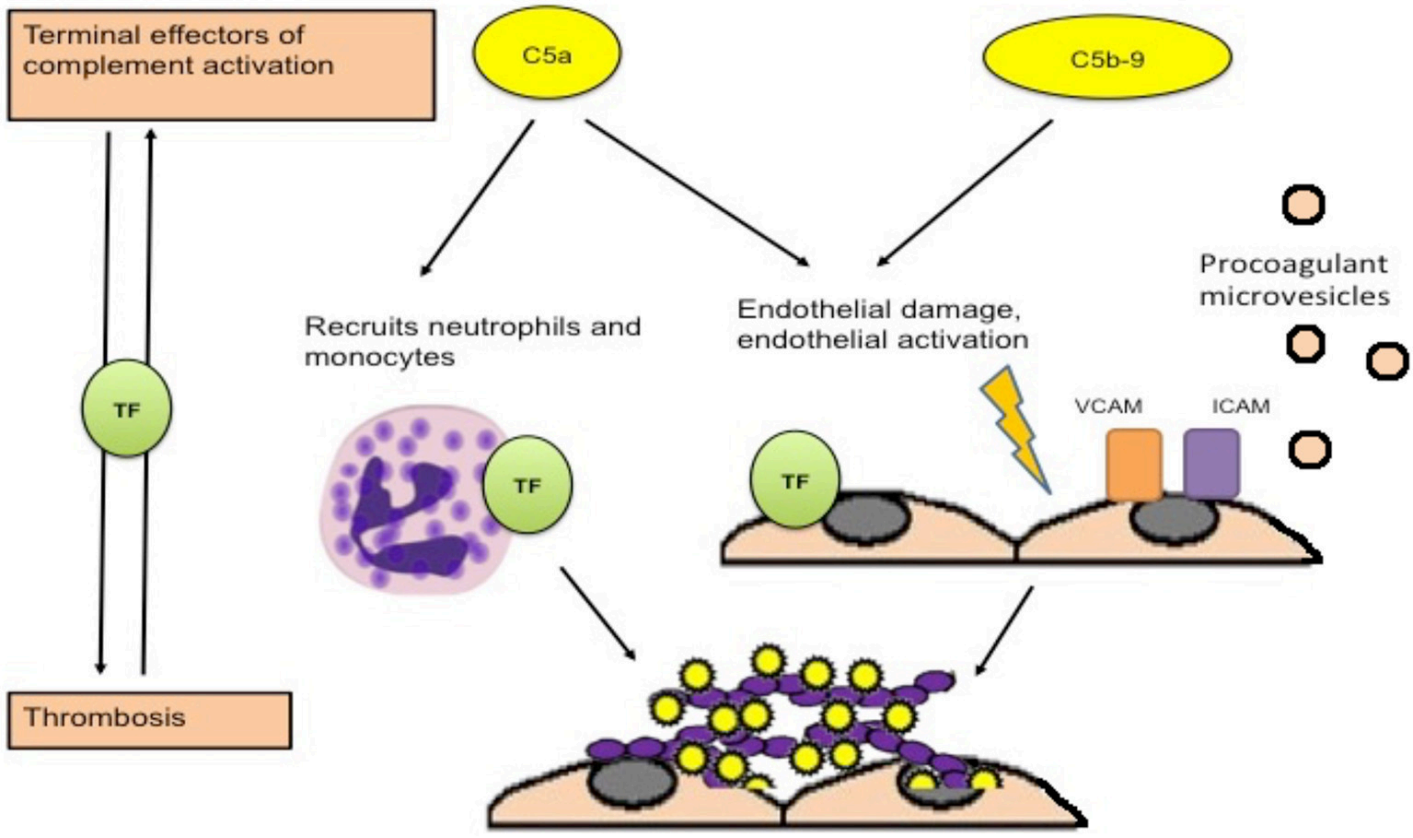
Procoagulant effects of complement activation. Activation of complement leads to generation of C5a and C5b, which combines with other terminal complement components to form the membrane attack complex. C5a is an anaphylatoxin that recruits neutrophils and leads to expression of tissue factor on neutrophils, monocytes and endothelial cells, which is associated with procoagulant activity. Deposition of the membrane attack complex on the endothelium leads to endothelial injury and procoagulant changes including expression of adhesion molecules, secretion of von Willebrand factor, and release of procoagulant microvesicles [adapted from Ritis et al. (26)].

Though the mechanisms of complement activation in APS are unclear, several mechanisms by which activated complement may contribute to thrombotic events have been suggested. Activation of complement leads to cleavage of C5, generating C5a and C5b (leading to membrane attack complex formation). Ritis et al. demonstrated that aPL-induced complement activation may lead to neutrophil expression of TF mediated through the C5a receptor, leading to expression of procoagulant activity (Figure 2) (26). C5a also induces TF expression on monocytes and endothelial cells (78, 79). In addition, deposition of C5b-9 on the endothelial surface leads to secretion of high molecular weight multimers of von Willebrand factor (80), expression of P selectin (81), and plasma membrane vesiculation that exposes a catalytic surface for the prothrombinase complex (82). Interestingly, a recent experiment by Müller-Calleja et al. indicated that a cofactor (β2GPI) -independent aPL induced exposure of procoagulant phosphatidylserine and activated TF on monocytes and induced thrombosis, and that C3 but not C5 was required (56). Complement activation can also contribute to depressed fibrinolysis, a recognized thrombogenic mechanism in APS (83–85). Events occurring during thrombosis and fibrinolysis can also activate complement leading to the generation of C5a and a functional membrane attack complex (86, 87), further amplifying activation of coagulation and thrombosis.

## Therapeutic Implications

Though long-term anticoagulation with vitamin K antagonists and a combination of aspirin with low molecular weight heparin are the mainstay of therapy for thrombotic and obstetric APS, respectively, some patients develop recurrent aPL-related clinical events despite “adequate” therapies, indicating a need for other treatments (52). The expanding data supporting a role of complement in aPL associated complications makes complement inhibition an attractive clinical target in APS. Moreover, complement targeted therapeutics are an active area of investigation, and the terminal complement inhibitor, eculizumab, is already widely used for the treatment of paroxysmal nocturnal hemoglobinuria and atypical hemolytic uremic syndrome. Although no complement inhibitors are approved for use in APS, eculizumab has been used successfully in patients with CAPS (54, 57, 61), to prevent recurrent CAPS in patients undergoing renal transplantation (88), and to prevent re-thrombosis in a patient with APS and recurrent arterial thrombosis (50). Recent reports of its successful use in CAPS (Table 1) are particularly encouraging since mortality in CAPS is as high as 40% with current treatment modalities (89). Pregnancy is considered a high-risk period for patients with aPL, with a high rate of fetal loss and pregnancy complications, and may serve as the “second hit” that leads to vascular complications. Preeclampsia and HELLP syndrome are also more common in women with aPL (55, 60), and may be complement mediated (90–92). Eculizumab crosses the placenta only minimally and does not affect the fetus (93). Hence, complement blockade may be an effective therapeutic modality for severe aPL related complications, including CAPS, during pregnancy (59, 94, 95).

Despite these encouraging data, there is still insufficient data to support the routine use of anti-complement therapy in APS, particularly patients without CAPS, and further mechanistic studies and randomized clinical trials are required. Ideally, complement-related biomarkers would be able to identify patients who are more likely to be refractory to standard therapy, and those who would benefit from complement inhibition as an adjunct to anticoagulation and antiplatelet therapy. However, standard measures of circulating complement cleavage products have not yet been shown to correlate with or predict the development of thrombosis.

## Conclusions

Recent experimental data indicate that complement activation plays a critical role in the pathogenesis of thrombosis and pregnancy complications in APS (25). However, the mechanisms by which aPL activate complement are not fully understood. Complement inhibition may provide a useful adjunctive therapy for patients with APS refractory to standard therapies, which is supported by reports of successful use of complement inhibition in patients with CAPS (54, 61). Mechanistic and clinical studies are needed to evaluate the efficacy of complement inhibition in APS and to develop biomarkers that can identify patients who might benefit from complement inhibition.

## Author Contributions

SC wrote the primary manuscript with the assistance and editing by KM and RB.

### Conflict of Interest Statement

The authors declare that the research was conducted in the absence of any commercial or financial relationships that could be construed as a potential conflict of interest.
